# Molecular surveillance of anti-malarial drug resistance in Democratic Republic of Congo: high variability of chloroquinoresistance and lack of amodiaquinoresistance

**DOI:** 10.1186/s12936-020-03192-x

**Published:** 2020-03-20

**Authors:** Doudou M. Yobi, Nadine K. Kayiba, Dieudonné M. Mvumbi, Raphael Boreux, Pius Z. Kabututu, Hippolyte N. T. Situakibanza, Joris L. Likwela, Patrick De Mol, Emile W. Okitolonda, Niko Speybroeck, Georges L. Mvumbi, Marie-Pierre Hayette

**Affiliations:** 1 Department of Basic Sciences, Faculty of Medicine, University of Kinshasa, Kinshasa, Democratic Republic of Congo; 2 School of Public Health, Faculty of Medicine, University of Kinshasa, Kinshasa, Democratic Republic of Congo; 3 School of Public Health & Research Institute of Health and Society, Catholic University of Louvain, 1200 Brussels, Belgium; 4 Laboratory of Clinical Microbiology, University of Liège, 4000 Liège, Belgium; 5 Department of Internal Medicine, Faculty of Medicine, University of Kinshasa, Kinshasa, Democratic Republic of Congo; 6 National Malaria Control Programme, Kinshasa, Democratic Republic of Congo

**Keywords:** Molecular, Surveillance, Resistance, Chloroquine, Amodiaquine, DRC

## Abstract

**Background:**

The loss of chloroquine (CQ) effectiveness has led to its withdrawal from national policies as a first-line treatment for uncomplicated malaria in several endemic countries, such as the Democratic Republic of Congo (DRC). The K76T mutation on the *pfcrt* gene has been identified as a marker of CQ resistance and the SVMNT haplotype in codons 72–76 on the same gene has been associated with resistance to amodiaquine (AQ). In the DRC, the prevalence of K76T has decreased from 100% in 2000 to 63.9% in 2014. The purpose of this study was to determine the prevalence of K76T mutations in circulating strains of *Plasmodium* *falciparum*, 16 years after CQ withdrawal in the DRC and to investigate the presence of the SVMNT haplotype.

**Methods:**

In 2017, ten geographical sites across the DRC were selected. Dried blood samples were collected from patients attending health centres. Malaria was first detected by a rapid diagnostic test (RDT) available on site (SD Bioline Malaria Ag *Pf* or CareStart Malaria *Pf*) or thick blood smear and then confirmed by a *P.* *falciparum* species-specific real-time PCR assay. A *pfcrt* gene segment containing a fragment that encodes amino acids at positions 72–76 was amplified by conventional PCR before sequencing.

**Results:**

A total of 1070 patients were enrolled. Of the 806 PCR-confirmed *P.* *falciparum* positive samples, 764 were successfully sequenced. The K76T mutation was detected in 218 samples (28.5%; 95% CI 25.4%–31.9%), mainly (96%) with the CVIET haplotype. Prevalence of CQ resistance marker was unequally distributed across the country, ranging from 1.5% in Fungurume to 89.5% in Katana. The SVMNT haplotype, related to AQ resistance, was not detected.

**Conclusion:**

Overall, the frequency of the *P.* *falciparum* CQ resistance marker has decreased significantly and no resistance marker to AQ was detected in the DRC in 2017. However, the between regions variability of CQ resistance remains high in the country. Further studies are needed for continuous monitoring of the CQ resistance level for its prospective re-use in malaria management. The absence of the AQ resistance marker is in line with the use of this drug in the current DRC malaria treatment policy.

## Background

*Plasmodium falciparum*, which is the most common and deadly *Plasmodium* species in sub-Saharan Africa, has developed resistance mechanisms to almost all existing anti-malarial drugs with a significant impact on malaria control. The World Health Organization (WHO) recommends monitoring the therapeutic efficacy of anti-malarials used in the treatment of uncomplicated *P.* *falciparum* malaria every 2 years. Beyond a 10% resistance rate, the WHO recommendation is to replace the drug with a more effective one [1]. The gradual decline in the therapeutic efficacy of chloroquine (CQ) has led to its withdrawal from the uncomplicated malaria treatment of *P.* *falciparum* in endemic countries. In the Democratic Republic of Congo (DRC), a clinical study conducted in 2001 to evaluate CQ effectiveness reported a treatment failure rate of 45.5% [2]. This high failure rate led to the replacement of CQ by the combination sulfadoxine-pyrimethamine (SP) in the management of uncomplicated malaria [2]. In 2005, due to the decreasing efficacy of the current treatments, the DRC adopted the use of artemisinin-based combination therapy (ACT) as the first-line treatment for uncomplicated malaria. Artesunate-amodiaquine (ASAQ) has been the mainstay of the country’s ACT anti-malarial drug policy since the implementation of ACT in the DRC. Artemether-lumefantrine (AL) was added as an alternative to ASAQ later in 2012. Both ASAQ and AL are now concurrently used in the treatment of uncomplicated *P falciparum* malaria in the DRC [3]. To explain clinical resistance to these anti-malarial drugs, molecular studies have been conducted and have identified mutations on several genes such as the *pfcrt* gene which encodes a membrane transporter protein located on the parasitic digestive vacuole in *P.* *falciparum* [4, 5]. The *pfcrt* gene is highly polymorphic, however the gene variant coding the PFCRT protein in which lysine (K) is replaced by threonine (T) at position 76 (K76T) is responsible for a lack of CQ accumulation in the digestive vacuole leading to resistance [5–9]. The majority of *pfcrt* gene mutations related to CQ resistance were localized between codons 72 and 76, determining different phenotypic profiles. CVMNK is the wild haplotype in CQ-sensitive parasites, whereas the CVIET, SVMNT, SVIET, CVMNT and CVTNT haplotypes are classically associated with CQ resistance [10]. The SVMNT haplotype was found to also be associated with resistance to amodiaquine (AQ), one of the components of some artemisinin-based combinations [11]. This haplotype has been reported in some neighbouring countries such as Tanzania and Angola, but not yet in the DRC [12, 13].

The prevalence of the K76T mutation has evolved considerably since the withdrawal of CQ from the management policy in several endemic countries. Studies have reported a decrease in the K76T mutation prevalence in some African countries such as Rwanda, the Republic of Congo and Kenya [14–17], whereas this prevalence remained high in other countries such as Ghana and Ethiopia several years after CQ withdrawal [18, 19]. The 2007 national Demographic Health Survey (DHS) reported a prevalence of 55.4% for the K76T mutation in the DRC [20]. This prevalence was 73.2% in a study conducted in Kinshasa in 2010 [21] and 63.9% in a study conducted in 2014 in 6 provinces of the DRC [22]. No marker of resistance to AQ has yet been reported in the DRC. The purpose of the present study was to determine the prevalence of the K76T mutation 16 years after CQ withdrawal from treatment policy and to investigate the presence of the SVMNT haplotype in the DRC.

## Methods

### Study area

**Fig. 1:**
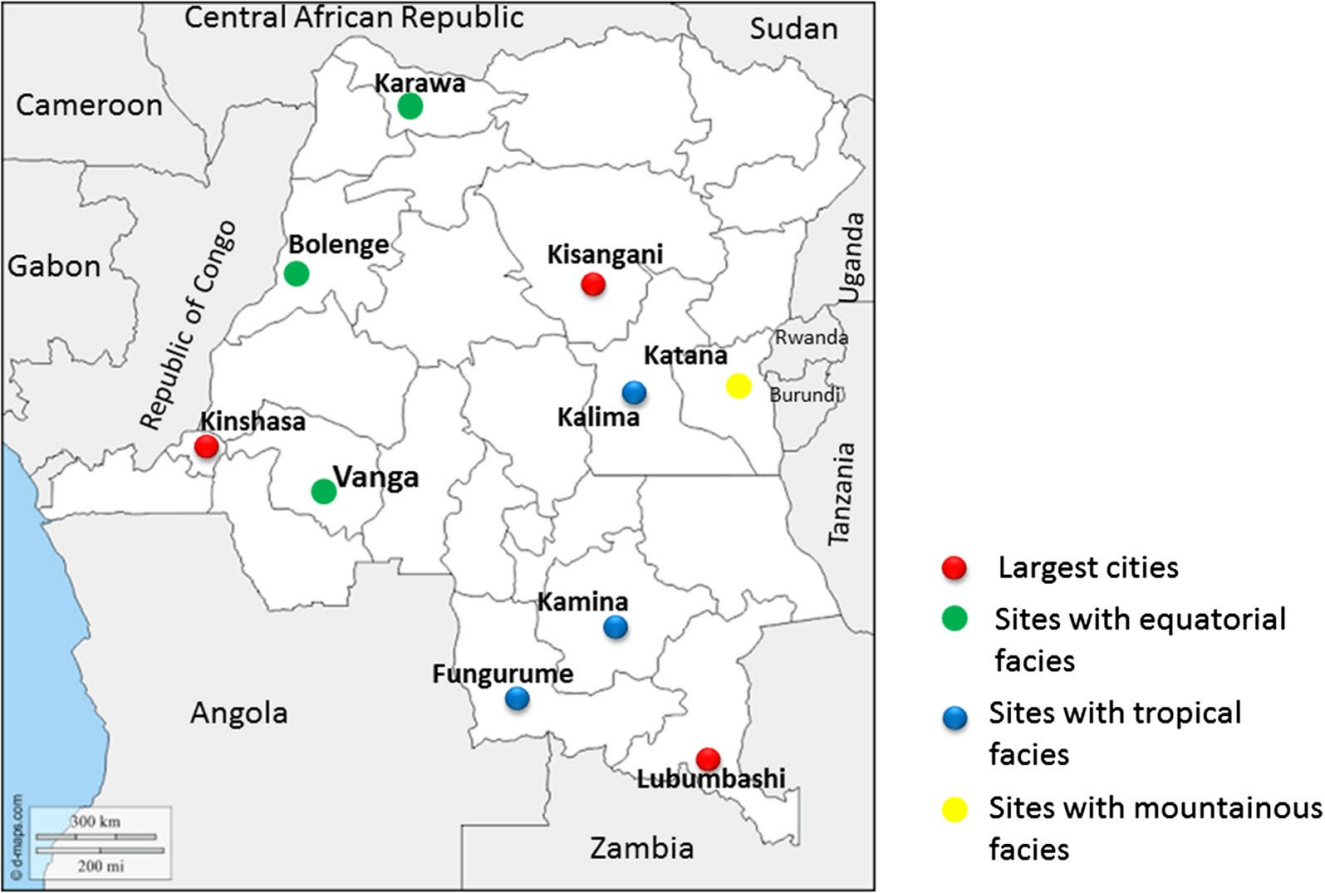
Map of study sites across the Democratic Republic of Congo (DRC)

The study was conducted in collaboration with the National Malaria Control Programme (NMCP). Ten sites were selected among the 26 provinces of the DRC. The study included the 3 largest cities of the country (Kinshasa, Kisangani and Lubumbashi) as well as 7 other sites that were selected based on their epidemiological facies, which refers to the malaria transmission intensities [23]. Thus, the following sites were selected: Bolenge, Karawa and Vanga for the equatorial facies characterized by high and permanent transmission; Kalima, Kamina and Fungurume for the tropical facies marked by seasonal variations with resurgences during the rainy season (8–9 months) and Katana for the mountainous facies with low transmission. Figure 1 presents a map of study sites according to the selection criteria. All collection sites are NMCP’s sentinel sites for malaria surveillance, except Lubumbashi. These sentinel sites are organized within one selected Health Zone entity, per province, and are part of a national network for malaria surveillance. The objectives of this network are to provide accurate, real-time data on morbidity-mortality trends, monitor the epidemiological progression for a rapid response, monitor and evaluate control interventions and progress towards the elimination of malaria. A pilot study was conducted first in Kinshasa from January to March 2017. Afterwards, the study was started in the other sites from September to December 2017.

### Study participants

Patients of all ages who attended one of the selected medical centres for fever and who had a positive rapid diagnostic test (RDT) for malaria or a positive thick blood smear were enrolled after informed consent was given.

### Blood sample collection

Screening tests were performed on blood samples taken by finger prick. RDTs were used depending on the availability of the test on site. Two RDTs were available which detected both *P.* *falciparum* HRP2 protein: the SD Bioline Malaria Ag Pf (Standard Diagnostics) assay was used to enroll patients attending all collection sites except in Kinshasa, where the CareStart Malaria Pf (Access Bio) assay was used in the majority of patients. In cases of a lack of RDTs, a positive thick blood smear test for malaria was performed for enrollment.

After enrollment, a blood sample was taken by finger prick and three spots were deposited on Whatman Grade GB003 filter paper (Whatman, GE Healthcare). Dried blood spots (DBS) were placed in an individual ziploc plastic bag containing silica gel desiccant and were then stored at room temperature before their transfer to the Clinical Microbiology Laboratory of the University of Liège for molecular analysis.

### DNA extraction

DNA was extracted from blood spots using the QIAamp DNA Mini Kit (Qiagen, Germany) following the recommended protocol for DBS. The extracted DNA was stored at – 20 °C before PCR testing.

### *Plasmodium* *falciparum* real-time PCR

A real-time PCR for the detection of *P.* *falciparum* was performed according to a modified procedure previously described [24]. Briefly, the mix contained 200 nM of *P.* *falciparum* primers and probe, a volume of 2.5 µl of Double-Dye Probe/Primer for Internal Positive Control (IPC), 2.5 µl of DNA virus culture (DIA-EIC/DNACy5) for IPC, 12.5 µl of 2× Taqman Universal PCR Master Mix (Applied Biosystems) and water to reach a total volume of 25 µl including 5 µl of DNA template. Assays were run on an ABI 7500 Fast real-time thermocycler (Applied Biosystems).

### *pfcrt* PCR

The fragment of interest (containing codons 72–76) on the *pfcrt* gene was amplified following a previously described procedure [21]. The PCR was run on a conventional Dyad Peltier Thermal Cycler (Bio-Rad Laboratories, CA, US). The PCR products were visualized after electrophoresis on 2% agarose gel stained with ethidium bromide.

### *pfcrt* genotyping

After purification using AMPure XP magnetic beads (Beckman Coulter, CA, US), the PCR products were added to a mix of Big Dye Terminator V3.1 for the sequencing reaction. The resulting 152-bp nucleotide sequences were analysed on an ABI 3730 DNA Analyzer automated sequencer (Applied Biosystems) using the Sanger method at GIGA, the interdisciplinary biomedical research centre of Liège University. These 152-bp nucleotide fragments of the *pfcrt* gene encompassing the codons at position 72–76, were aligned using GeneStudio™ Professional and compared to the reference sequence PF3D7_0709000 (https://www.ncbi.nlm.nih.gov/gene/term=PF3D7_0709000 accessed on September 11, 2018) using the online Basic Local Alignment Search Tool (BLAST) for identifying mutations.

### Ethical considerations

The protocol and the informed consent form were approved by the Ethics Committee of the Faculty of Medicine, University of Kinshasa (Approval No: ESP MINESU 019/2016). All participants involved in the study signed an informed consent form. Where participants were young (children), the consent form was approved and signed by their parents or guardians.

### Statistical analysis

Data were entered in an Excel 2010 database by an independent data clerk. Statistical analysis was performed using SPSS V. 20.0 (IBM corp., Armonk, NY). The *pfcrt* genotype profile was determined by the absence or presence of wild/mutant alleles. Samples for which the genotype profile could not be determined were excluded from the analysis. Difference between sites was assessed using the Chi square test for proportions and a *p* value of less than 0.05 was considered statistically significant.

## Results

In total, 1070 patients were enrolled in the study, their age ranged from 0 to 74 years. Table 1 presents the distribution of patients per age range: children aged from 0 to 5 years were the most affected age category.

**Table 1:** Distribution of study patients per age range

Age range (year)	Effective	Frequency % (95% CI)
0–5	465	43.5 (40.5–46.5)
6–14	281	26.3 (23.7–29.0)
15–49	285	26.6 (24.0–29.4)
50–74	39	3.6 (2.6–5.0)
Total	1070	100

**Fig. 2:**
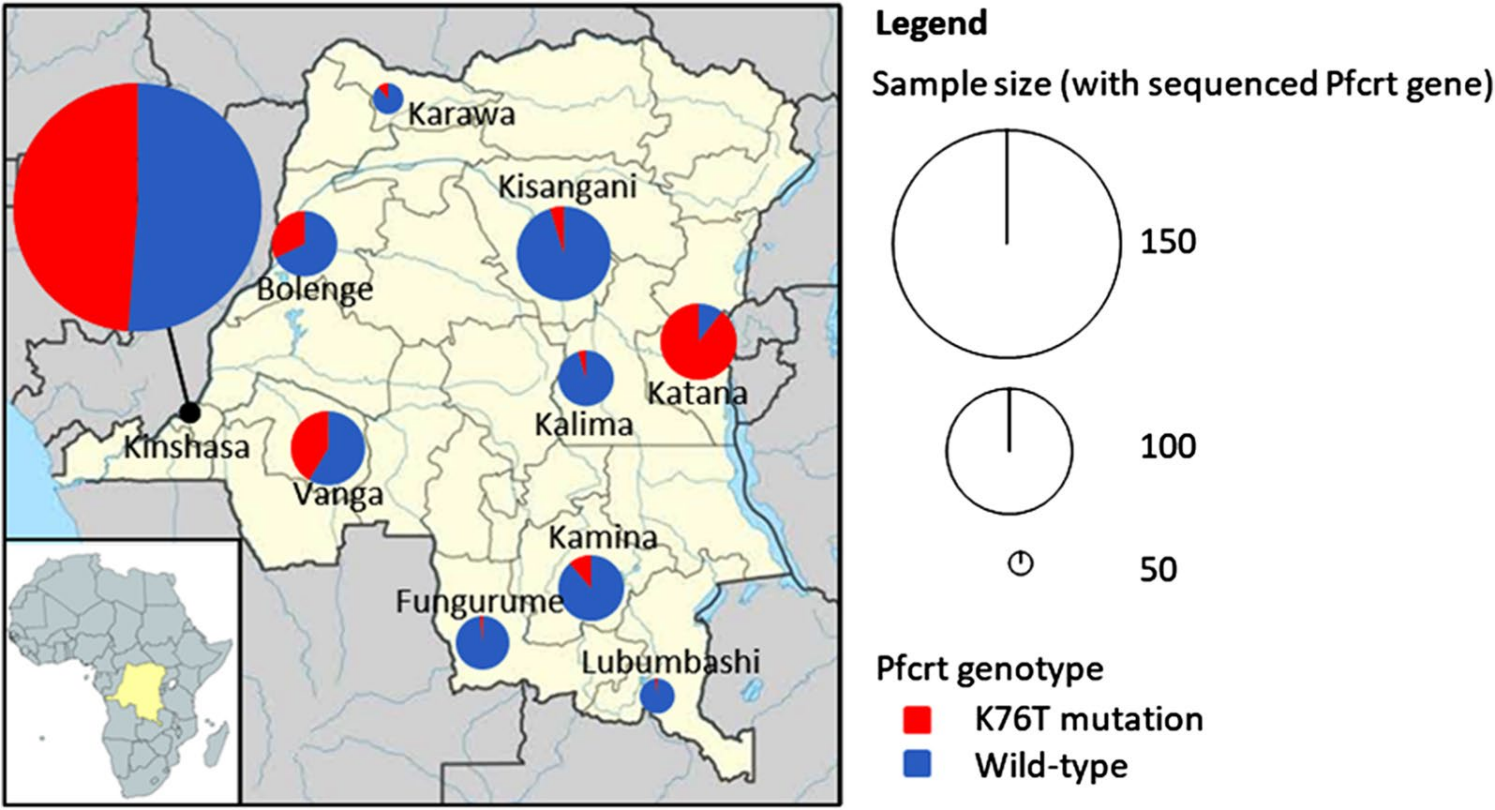
Geographical distribution of K76T mutation rate across the DRC

Real-time PCR analysis of DNA extracted from DBS samples confirmed the initial diagnosis of *P.* *falciparum* infection for 806 (75.3%) patients. Of the 764 successfully sequenced *P.* *falciparum* isolates, 218 (28.5%) carried the K76T mutation known to confer resistance to CQ. Table 2 reports the prevalence of K76T mutations in different collection sites in the DRC. The highest rate (89.5%) was detected in Katana, in the eastern province of Sud-Kivu, and the lowest rate (1.5%) in Fungurume, in the southeastern province of Lwalaba. Globally, a significant difference was observed between all study sites (p < 0.001). Figure 2 shows the distribution of K76T mutations across the DRC. Four out of ten sites (Kinshasa, Bolenge, Vanga and Katana) showed a K76T prevalence rate higher than 30%.

**Table 2:** Prevalence of K76T mutation per study site

Site	Positive RDT/thick blood sample N	Positive *Pf* PCR N (%)	Samples successfully sequenced among positive *Pf* PCR N	Samples with K76T mutant N	Prevalence of K76T mutation % (95% CI)
Bolenge	98	73 (74.5)	56	18	32.1 (20.3–46.6)
Fungurume	92	65 (70.7)	65	1	1.5 (0.0–8.3)
Kalima	97	67 (69.1)	66	3	4.5 (0.9–12.7)
Kamina	98	73 (74.5)	71	8	11.3 (5.0–21.0)
Karawa	97	66 (68.0)	54	6	11.1 (4.2–22.6)
Katana	105	78 (74.3)	76	68	89.5 (80.3–95.3)
Kinshasa	160	160 (100)	160	78	48.8 (40.8–56.8)
Kisangani	128	93 (72.7)	85	4	4.7 (1.3–11.6)
Lubumbashi	101	56 (55.4)	56	1	1.8 (0.0–9.6)
Vanga	94	75 (79.8)	75	31	41.3 (30.1–53.3)
Total	1070	806 (75.3)	764	218	28.5 (25.4–31.9)

*N* number; globally, there was a significant difference of K76T prevalence between sites (p < 0.001)

Table 3 shows the CQ-resistant PFCRT 72–76 haplotypes that were detected in each study site; the CVIET haplotype was predominant in isolates carrying K76T mutations (95.9%). The CQ-sensitive CVMNK haplotype was found in wild-type *P.* *falciparum* isolates, while the SVMNT haplotype associated with AQ resistance was not detected in the present study.

**Table 3:** Repartition of the CQ-resistant PFCRT 72–76 haplotypes in the different sampling sites

Study site	Sample with K76T mutant N	CVIET haplotype N (%)	GVIET haplotype N (%)	CVMET haplotype N (%)
Bolenge	18	18 (100)	0 (0.0)	0 (0.0)
Fungurume	1	1 (100)	0 (0.0)	0 (0.0)
Kalima	3	3 (100)	0 (0.0)	0 (0.0)
Kamina	8	8 (100)	0 (0.0)	0 (0.0)
Karawa	6	6 (100)	0 (0.0)	0 (0.0)
Katana	68	66 (97.0)	1 (1.5)	1 (1.5)
Kinshasa	78	76 (97.4)	0 (0.0)	2 (2.6)
Kisangani	4	3 (75.0)	0 (0.0)	1 (25.0)
Lubumbashi	1	0 (0.0)	1 (100)	0 (0.0)
Vanga	31	28 (90.3)	2 (6.5)	1 (3.2)
Total	218	*209 (95.9)*	4 (1.8)	5 (2.3)

## Discussion

This study was conducted in ten different sites across the DRC for monitoring molecular resistance of *P.* *falciparum* to CQ and to AQ. The study has shown that 16 years after official CQ withdrawal from the national treatment policy, CQ-resistance prevalence has decreased in DRC to 28.5% and is marked by a high variability from site to site that was found to be significantly different (p < 0.001). No molecular marker of AQ resistance has been found.

A decline in the prevalence of resistance has also been reported in several other African countries. An in vivo assay conducted in 2005 [25] revealed a return of CQ efficacy to 99% in Malawi, the first country to change its national first-line malaria treatment policy from CQ to SP in 1993. More studies have shown the return of efficacy for CQ several years after its withdrawal from treatment policies. In Tanzania, CQ resistance decreased from 80% in 2001 to 5.7% in 2011 [26]. In Kenya, it decreased from 76 to 6% between 2003 and 2015 [27]. In the Republic of Congo (Brazzaville), resistance decreased from 100% in 2005, 1 year before the introduction and implementation of ACT in 2006, to 71% in 2015 [14]. In Zambia, no CQ resistance marker was detected in a clinical trial conducted from 2010 to 2013 [28].

The relationship between drug pressure and CQ sensitivity has been clearly reported by Feng et al. and Frosch et al. [16, 29]. When the pressure stops, the drug tends to recover its effectiveness against the parasite. Mutations are common in parasites. However, the fitness of the mutant, which is its ability to survive compared to the wild-type parasite, may be altered as shown by a deficit of reproductive potential in a number of mutants that are otherwise viable [30]. Consequently, the majority of mutant parasites will gradually disappear from the natural population, allowing the emergence of the wild-type population [30]. This explanation could partly explain the observations of a very low prevalence of K76T mutations detected in Fungurume (1.5%), Lubumbashi (1.8%), Kalima (4.5%) and Kisangani (4.7%) in the present study. However, this prevalence was higher in other sites such as Bolenge (32%), Vanga (41.3%), Kinshasa (48.8%) and especially at Katana (89.5%). Previous studies have reported much higher K76T rates in Kinshasa in 2008 (83.8%) [31] and 2010 (73.2%) [21], and in Bolenge in 2014 (70.6%) [22].

The simultaneous presence of very low and high prevalence of CQ resistance could be related to different levels of CQ pressure between study sites, differing from one site to another, before and after the withdrawal of this molecule. Concerning the use of CQ in DRC, data from the DHS II in 2013–2014 revealed that in provinces where our sites are located (before territorial apportionment from 11 to 26 provinces) CQ was still in use despite its withdrawal from the national policy of malaria management in 2001 [32]. Unfortunately, these DHS data have not yet been updated in the 26 current provinces and the low remaining use of CQ cannot fully explain the disparity of CQ resistance observed in different locations. Thus, further studies at the community level should be conducted to enrich the data.

The Katana site which had the highest rate of CQ resistance (89.5%) is located in the eastern province of Sud-Kivu, where several armed militias have been warring during the last two decades. This instability in the country’s security is an obstacle to good management of the use of anti-malarial drugs as recommended by the national malaria treatment policy. Lack of control of the anti-malarial supply chain could result in the use of non-recommended molecules by the population, such as CQ. In contrast, other sites such as Lubumbashi in the southeast of the country, where the security situation has been calmer, have seen a significant decrease in the prevalence of the K76T mutation in parasites. Another distinct feature of Katana is its epidemiological facies. The Katana site corresponds to the mountainous facies, where a lower malaria transmission rate results in a low rate of sexual recombination of parasite genotypes in the mosquito. As a consequence, the drug resistant genotypes become more established in the host population. However, Bushman et al. have recently suggested an alternative hypothesis based on the competition between drug-sensitive and drug-resistant parasites within the human host. This competition could slow the spread of resistance in high-transmission settings, which are marked by mixed parasite strain and genotype infections [33]. Conversely, human malaria infections consisting of multiple parasite genotypes are rarely observed in low transmission settings. Drug resistance appears and emerges from this kind of place; as previously shown in Southeast Asia, considered to be the bastion of anti-malarial resistance [30]. In Africa, anti-malarial drug resistance has historically risen in the east and spreads to the rest of the continent [34]. As a reminder, the first case of resistance to CQ was reported in Tanzania [35]. The province of Kivu in the eastern Congo (where Katana is located) was the first region to report CQ resistance in the DRC [36]. In addition, a therapeutic efficacy study conducted in 2001 in the DRC, found that the city of Bukavu in Kivu contained the highest percentage (80%) of patients who had treatment failure with CQ [2]. This finding shows a high conservation of the K76T mutation in a part of the DRC 16 years after the discontinuance of CQ as first-line therapy in the DRC national malaria policy. Future molecular studies are necessary to monitor the trends in CQ resistance marker rates and to confirm or refute this disparity across the country.

Concerning *pfcrt* haplotypes, the CVIET haplotype was predominant in isolates carrying K76T mutations in this study. However, there are many other possible combinations of polymorphisms in positions 72–76 that include the key mutation K76T in CQ-resistant *P.* *falciparum,* with CVIET as the most common haplotype in Africa [10]. The single mutation in codon 76 is rarely observed in nature, suggesting that compensatory mutations in codon positions other than 76 could be required to restore the fitness of the CQ-resistant parasites bearing the K76T mutation [10]. The SVMNT haplotype associated with AQ resistance was not detected in the present study which is good news for ACT use. This haplotype has not yet been reported in the DRC [20–22] whereas it was found in neighbouring countries, such as Tanzania and Angola [12, 13]. Regular monitoring of resistance to AQ is required because AQ is the partner molecule in ASAQ combination therapy, which is one of the artemisinin-based combinations currently used in the country.

Out of 806 positive *P.* *falciparum* samples sequenced, 42 samples (5.2%) have not given interpretable sequences. The yield of sequencing depended on several factors including the concentration of template used in the reaction. The discordance between screening test results (RDTs and thick blood test) and those of PCR *P.* *falciparum* detection will be addressed in a future publication.

## Limitation

The limitation of the study is the low sample size (not representative of all the country) that results in a partial explanation of the disparity in the CQ resistance marker rate between the study sites.

## Conclusion

Overall, the frequency of the *P.* *falciparum* CQ resistance marker has decreased significantly and no resistance marker to AQ was detected in the DRC. However, the between regions variability of CQ resistance remains high in the country. Further studies are needed for continuous monitoring of the CQ resistance level for its prospective re-use in malaria management. The absence of AQ resistance is in line with the use of this drug in the current DRC malaria treatment policy.

## Data Availability

All data generated and analysed during this study are included in this published article.

## Abbreviations

WHO: World Health Organization
DRC: Democratic Republic of Congo
*pfcrt*: *Plasmodium falciparum* chloroquine resistance transporter
CQ: Chloroquine
AQ: Amodiaquine
ACT: Artemisinin-based combination therapy
DNA: Deoxyribonucleic acid
PCR: Polymerase chain reaction
RDT: Rapid diagnostic test
SP: Sulfadoxine + Pyrimethamine
ASAQ: Artesunate + Amodiaquine
AL: Artemether + Lumefantrine
